# N6-methyladenosine-modified GPX2 impacts cancer cell stemness and TKI resistance through regulating of redox metabolism

**DOI:** 10.1038/s41419-025-07764-0

**Published:** 2025-06-18

**Authors:** Xu Yang, Long Yu, Miaomiao Shao, Huiling Yang, Kangwei Qi, Gaofei He, Lanxin Wang, Di Kong, Jianxin Gu, Xiaolin Xu, Lan Wang

**Affiliations:** 1 NHC Key Laboratory of Glycoconjugate Research, Department of Biochemistry and Molecular Biology, School of Basic Medical Sciences, Fudan University, Shanghai, 200032 China; 2 School of Medicine, Nanjing University of Chinese Medicine, Nanjing, China; 3 Department of Pathology, The First Affiliated Hospital of Naval Medical University, Shanghai, 200003 China; 4 Department of Cardiothoracic Surgery, The Third Affiliated Hospital of Naval Medical University, Shanghai, 200003 China

**Keywords:** Cancer microenvironment, Mechanisms of disease, Cancer stem cells

## Abstract

As a predominant oncogenic driver in non-small cell lung cancer (NSCLC), EGFR frequently undergoes amplification or mutation, with EGFR-tyrosine kinase inhibitors (EGFR-TKIs) like gefitinib and erlotinib constituting frontline therapy for advanced EGFR-mutant cases. However, both primary and acquired resistance significantly limit clinical efficacy. Here, we revealed that glutathione metabolic pathway controlled by glutathione peroxidase GPX2 was abnormally activated in gefitinib-resistant A549 and HCC827-GR cell lines. Mechanistically, GPX2 triggers Hedgehog signaling activation through releasing GLI transcriptional regulator, promoting cancer stem cell (CSC) characteristics and TKI resistance. Notably, N6-methyladenosine (m^6^A) modification on GPX2 mRNA mediated by METTL14 diminished its stability. In vivo, GPX2 deletion constrained glutathione metabolism and boosted the effectiveness of TKI in cell line-derived xenograft models. Collectively, these findings demonstrate that GPX2 serves as a positive regulator of both primary and acquired EGFR-TKI resistance and could be a promising therapeutic target for precise treatment of NSCLC.

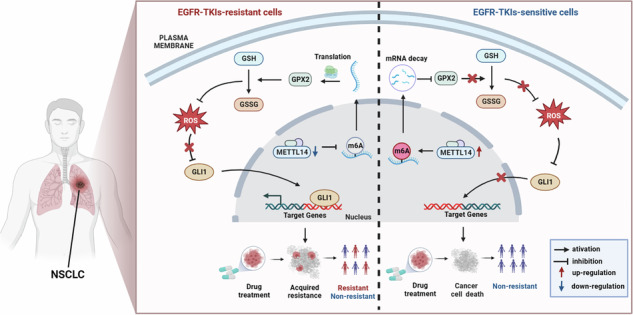

## Introduction

The epidermal growth factor receptor (EGFR), an important transmembrane tyrosine kinase receptor, is often overexpressed or mutated in non-small cell lung cancer (NSCLC), which accounts for 85% of lung cancer [1]. EGFR-targeted tyrosine kinase inhibitors (TKIs), particularly first-generation agents like gefitinib, have significantly advanced NSCLC treatment [2]. Third-generation EGFR-TKI osimertinib, developed to overcome T790M mutation-driven resistance, is now a first-line option for advanced EGFR-mutant NSCLC [3, 4]. However, third-generation EGFR-TKI remains a challenge, with resistance mechanisms including EGFR-dependent and non-EGFR-dependent resistance [5, 6], such as the C797S mutation [7, 8], MET amplification [9, 10], HER2 mutation [11], or amplification, etc. In addition to the inevitable acquired resistance, current EGFR-TKIs primarily benefit patients with EGFR-activating mutations, offering limited efficacy for wild-type EGFR (WT-EGFR) lung cancer patients. However, a growing body of evidence suggests that WT-EGFR is critical in lung cancer progression, linked to third-generation EGFR-TKI resistance and KRAS-driven NSCLC maintenance [11–13]. Thus, identifying novel mechanisms for targeting both mutated and WT-EGFR is imperative to enhance therapeutic strategies for NSCLC.

Mild reactive oxygen species (ROS) generation has been implicated in cancer development and progression via triggering DNA mutation and pro-tumorigenic signaling. The glutathione (GSH) redox system is one of the most critical antioxidant defense mechanisms in the body, shielding cells from ROS-induced damage. As the most abundant endogenous antioxidant, GSH can react with hydrogen peroxide or lipid peroxide to eliminate excess ROS [14]. Loss of GSH will destroy the redox homeostasis and increase ROS accumulation, leading to oxidative damage and cell death. It is reported that the basal ROS level was enhanced in EGFR-TKI resistant NSCLC cells, and the increased ROS induced upregulation of antioxidation procedure to restore redox homeostasis [15]. Therefore, targeting the GSH redox system holds great potential as a combination therapy for medical interventions to surmount tumor progression and drug resistance.

Glutathione peroxidases (GPXs), key antioxidant enzymes in GSH redox metabolism, comprise eight isoforms (GPX1-8), all of which catalyze hydroperoxide reduction using GSH as a reductant [16]. Selenium-dependent GPX isoforms include GPX1-4 and GPX6, whose mRNA stability declines under selenium deficiency [17]. GPX2, prioritized in selenoprotein biosynthesis, exhibits the greatest mRNA stability among GPXs during selenium restriction, underscoring its unique physiopathological role. GPX2 is specifically expressed in epithelial cells, mainly in the gastrointestinal tract, but the organ specificity will be lost when normal cells are transformed into cancer cells [18]. GPX2 can mediate ROS clearance for metabolic reprogramming and alter signaling pathways to affect tumor development [19, 20]. In NSCLC, GPX2 promotes epithelial-mesenchymal transition (EMT) and metastasis by activating the PI3K/AKT/mTOR/Snail signaling axis through the clearance of ROS [21], but the function of GPX2 on EGFR-TKI resistance remains unclear.

## Results

### Glutathione metabolism is elevated in EGFR-TKI resistant NSCLC cells

**Fig. 1:**
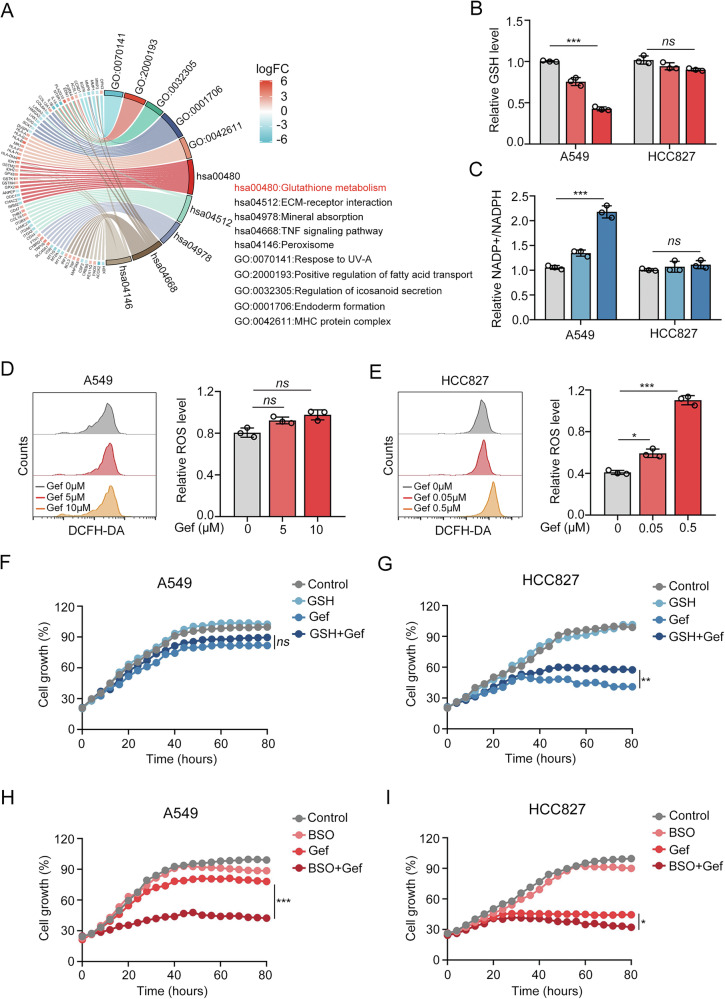
Glutathione metabolism is elevated in EGFR-TKI resistant NSCLC cells. **A** Chord plot analysis of GO terms and KEGG pathways between gefitinib-resistant and gefitinib-sensitive cells. Relative GSH level (**B**) and NADP + /NADPH ratio (**C**) of A549 and HCC827 cells treated with or without gefitinib. (A549: 0 μM, 5 μM, 10 μM; HCC827: 0 μM, 0.05 μM, 0.5 μM). ROS production in gefitinib-treated A549 (**D**) and HCC827 (**E**) cells. The effect of GSH supplementation on gefitinib sensitivity in A549 (**F**) and HCC827 (**G**) cells was monitored continuously by ECIS model 9600. The effect of buthionine sulfoximine (BSO) treatment on gefitinib sensitivity in A549 (**H**) and HCC827 (**I**) cells. *P < 0.05, **P < 0.01, ***P < 0.001, ns no significance.

For exploring the main cause of drug resistance in EGFR-TKI therapy, we performed GO and KEGG enrichment analysis of differentially expressed genes between gefitinib-resistant and sensitive cells from GEO database GSE103155. As shown in Fig. 1A, four upregulated pathways were identified, including glutathione metabolism, positive regulation of fatty acid transport, peroxisome and MHC protein complex. Among these pathways, genes associated with glutathione metabolism demonstrated the highest degree of enrichment. Glutathione system operates antioxidant defense by converting GSH to GSSG, while NADPH is consumed to maintain the intracellular GSH balance [22]. We next treated gefitinib sensitive cells HCC827 and gefitinib resistant cells A549 with different concentrations of gefitinib for 24 h and detected changes in reducing equivalents (GSH, NADPH). The results showed a decrease of GSH level and an increase of NADP + /NADPH ratio in gefitinib-resistant A549 cells, but there were no significant changes in gefitinib-sensitive HCC827 cells (Fig. 1B–C). 2’,7’-Dichlorodihydrofluorescein diacetate (DCFH-DA) probe detection indicated that there was no obvious change in ROS level of A549 cells after gefitinib treatment, while in HCC827 cells, ROS level raised substantially with the incremental drug concentration (Fig. 1D–E). These results suggest that gefitinib-resistant cells could relieve oxidative damage better. To investigate the role of glutathione metabolism in gefitinib resistance, we utilized the Electric Cell-substrate Impedance Sensing (ECIS) model to assess the effect of glutathione (GSH) compensation on cell growth dynamics. Oddly, GSH supplementation had no effect on A549 cell lines but enhanced the resistance of HCC827 cell lines to gefitinib mildly (Fig. 1F–G). Meanwhile, GSH biosynthesis inhibitor buthionine sulfoximine (BSO) significantly reversed drug resistance of A549 cell lines but had a slight influence on HCC827 cell lines (Fig. 1H–I). Collectively, our findings demonstrate that the glutathione metabolism pathway regulates cellular redox balance in TKI-resistant cells by enhancing GSH utilization and NADPH regeneration, thereby driving gefitinib resistance.

### Silencing GPX2 expression mitigates EGFR-TKI resistance of cancer cells

**Fig. 2:**
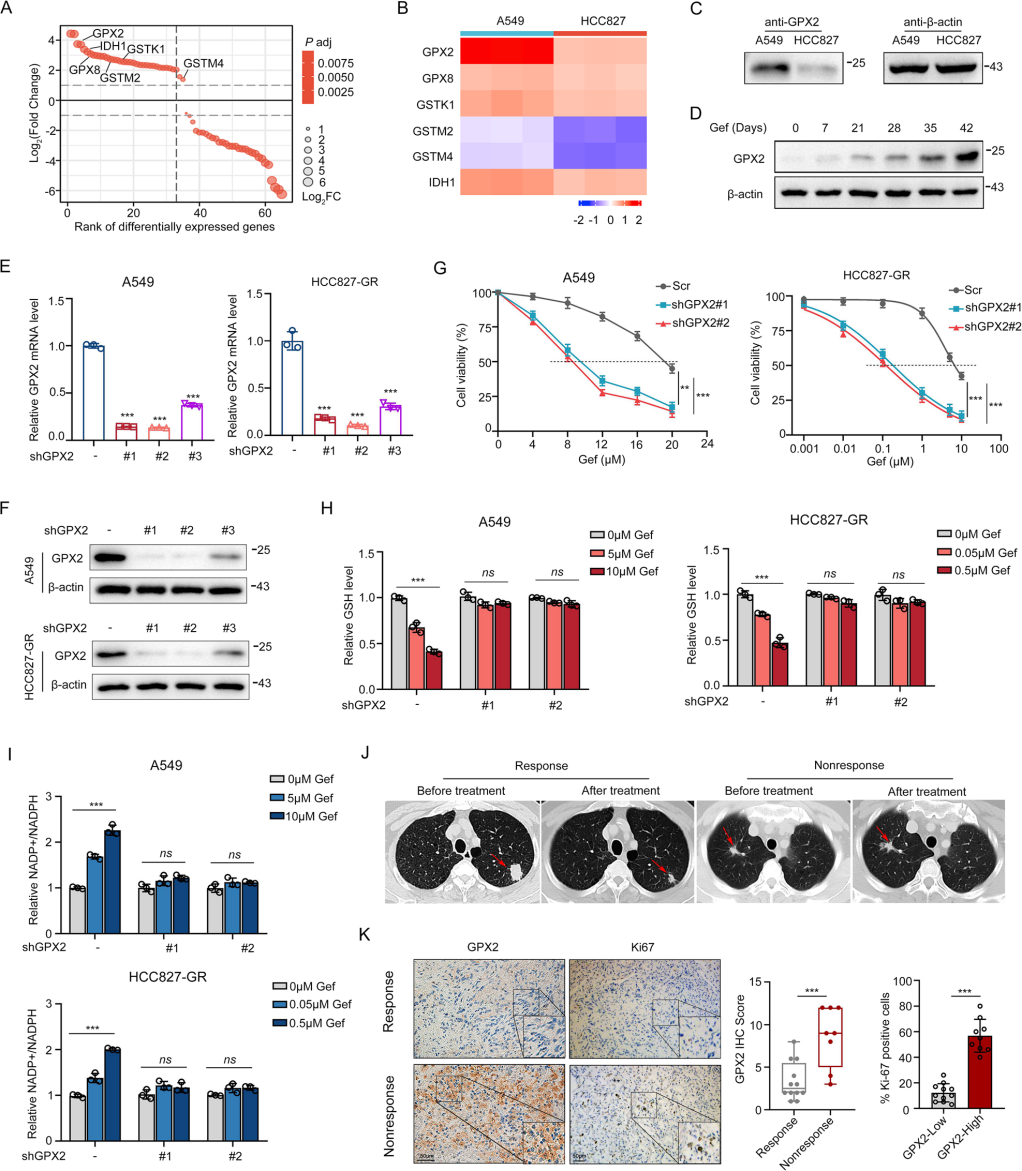
Loss of GPX2 mitigates EGFR-TKI resistance. **A** Differential gene analysis in the glutathione metabolic pathway of gefitinib-resistant and sensitive cells. **B** Heatmap illustrating alterations in mRNA level of the enzymes related to glutathione metabolic pathway in A549 compared to HCC827 cells. **C** The protein expression of GPX2 in A549 and HCC827 cells. **D** GPX2 protein levels at days 0, 7, 21, 28, 35 and 42 during the construction of HCC827-GR cells. Knockdown efficiency of GPX2 at mRNA (**E**) and protein level (**F**) in A549 and HCC827-GR cells. **G** IC50 measurement of gefitinib after scramble or GPX2 shRNA was stably transfected into A549 and HCC827-GR cells. **H** Measurement of GSH level in A549 and HCC827-GR cells transfected with scramble or GPX2 shRNA at different concentrations of gefitinib treatment. **I** NADP + /NADPH ratio was measured in A549 and HCC827-GR cells transfected with scramble or GPX2 shRNA under gefitinib treatment. **J** Representative computed tomography imaging with TKI-responsive or non-responsive lung cancer patients before and after treatment. Red arrows represent the primary tumor. **K** IHC staining of GPX2 expression and Ki67 expression in TKI-response or nonresponse lung cancer tissues. Scale bar, 50 µm. **P < 0.01, ***P < 0.001, ns no significance.

Given that glutathione metabolism involved multiple catalytic reactions controlled by a series of enzymes, we screened six significantly up-regulated gene-encoded enzymes related to glutathione metabolic pathway from GEO database GSE103155 (Fig. 2A). Further mRNA analysis of those enzymes in HCC827 and A549 cells revealed that peroxidase GPX2 was up-regulated most in A549 cells (Fig. 2B). Consistent with this, the protein level of GPX2 in A549 cells was also much higher than HCC827 cells (Fig. 2C). Enthrallingly, during the process of gefitinib-resistant HCC827 (HCC827-GR) cell lines construction, the protein level of GPX2 gradually increased along with the prolonged gefitinib exposure time. (Fig. 2D), suggesting that GPX2 may play a crucial role in EGFR-TKI resistance.

To substantiate the involvement of GPX2 in glutathione metabolism-dependent drug resistance, we established GPX2 shRNA knockdown stable cell lines, including A549 and HCC827-GR cell lines (Fig. 2E–F). Cell viability analysis showed that the IC50 of gefitinib significantly decreased in GPX2 deficient cells (Fig. 2G). Our prior results observed that gefitinib reduced GSH levels and elevated the NADP + /NADPH ratio in TKI-resistant cells (Fig. 1B–C), prompting us to explore whether GPX2 plays a critical role in gefitinib-stimulated GSH efflux. As shown in Fig. 2H–I, loss of GPX2 in A549 and HCC827-GR cells impeded the gefitinib-induced decline in GSH level and rise in NADP + /NADPH ratio. To assess the clinical relevance of GPX2 expression in gefitinib response, we collected tumor tissues from lung cancer patients undergoing TKI therapy. Patients were clinically categorized into TKI-responsive and TKI-nonresponsive groups based on computed tomography (CT)-assessed tumor burden (Fig. 2J). Immunohistochemical (IHC) staining revealed that TKI-nonresponse specimens from patients with lung cancer exhibited more intense IHC staining of GPX2 compared to TKI-response group (Fig. 2K). Notably, the percentage of Ki-67 positive area in GPX2-high expression group is also larger than GPX2-low expression group (Fig. 2K), implying that low GPX2 level in lung cancer may serve as an evaluating indicator for TKI response. Collectively, our results demonstrate that GPX2 inhibition enhances the sensitivity of drug-resistant cells and synergistically overcomes TKI resistance when combined with EGFR-TKI treatment.

### GPX2 enhances CSC characteristics in EGFR-TKI resistant cells

**Fig. 3:**
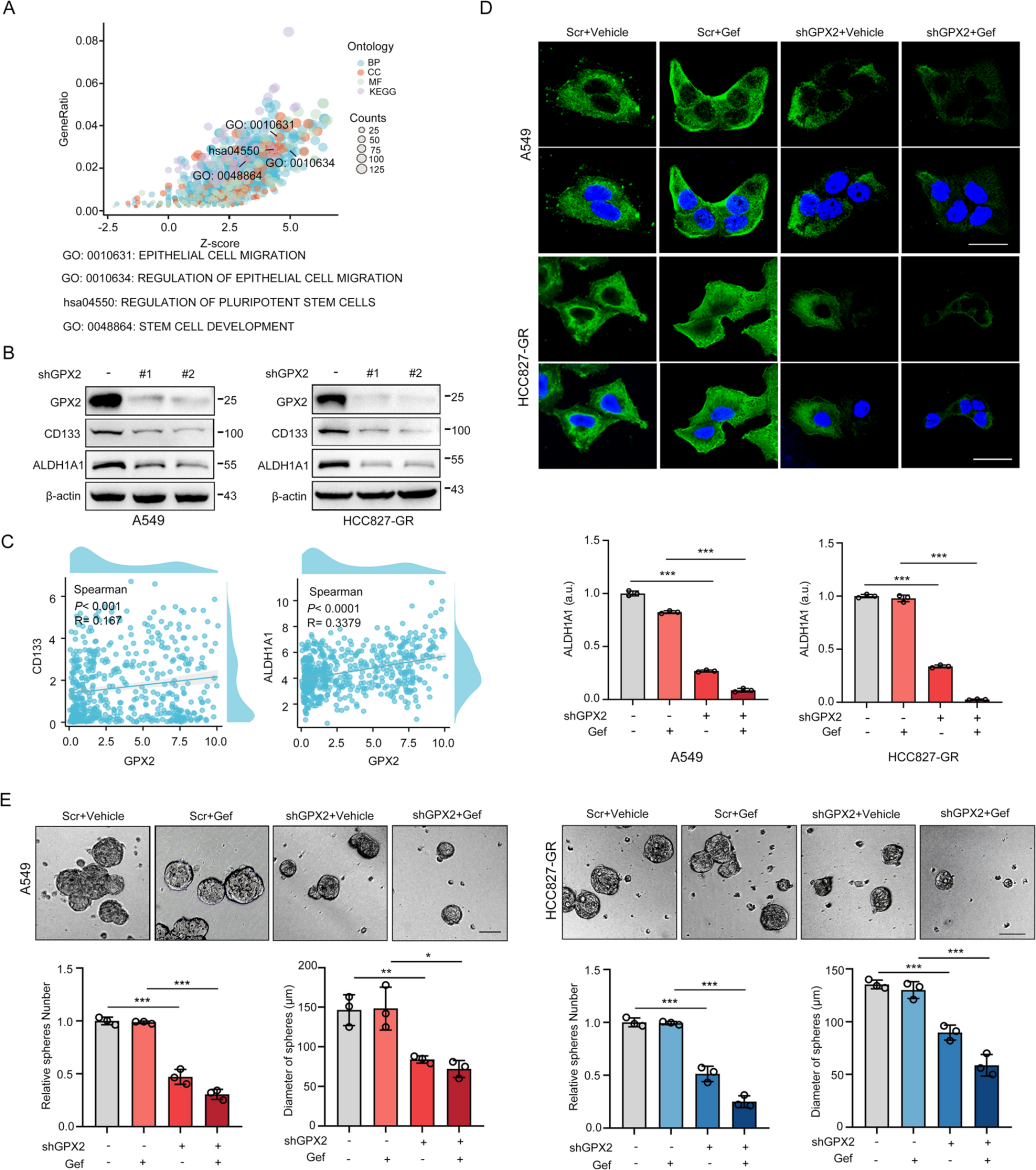
GPX2 enhances CSC characteristics in EGFR-TKI resistant cells. **A** Bubble plot depicting GSEA analysis of gene sets in lung cancer tissues with high or low GPX2 expression from TCGA database. **B** CD133 and ALDH1A1 expression levels in TKI-resistant cells after GPX2 depletion. **C** Scatter plot showing the correlation between GPX2 and CD133 or ALDH1A1 in the TCGA dataset. **D** Immunofluorescence detection of ALDH1A1 (green) expression in A549 and HCC827-GR cells. Scale bar, 20 µm. **E** Sphere formation ability of gefitinib-treated A549 and HCC827-GR cells under GPX2 knockdown. Scale bar, 100 µm. *P < 0.05, **P < 0.01, and ***P < 0.001, ns no significance.

Cancer stem cells (CSCs) represent a heterogeneous population of intra-tumor cells with high tumorigenic capacity and confer anticancer therapy resistance [23]. Especially, the GSEA enrichment analysis from the GEO database (GSE40791) revealed that GPX2 was associated with stem cell features (Fig. 3A). We then silenced GPX2 expression in EGFR-TKI resistant cells and analyzed the level of stemness markers. In comparison to cells expressing a scrambled shRNA (Scr), GPX2 deficient cells showed significantly decreased CD133 and ALDH1A1 levels (Fig. 3B). Similarly, gene expressional analysis in lung cancer from TCGA database revealed a positive correlation between GPX2 and CD133, as well as ALDH1A1 (Fig. 3C). To investigate the influence of GPX2 combination with gefitinib on cell stemness, we quantified ALDH1A1 expression profiles by immunofluorescence staining. The data demonstrated that ALDH1A1 level was declined much more in GPX2 deficient cells with gefitinib treatment, as compared to those without gefitinib treatment (Fig. 3D). Additionally, sphere formation assay displayed that GPX2 knockdown alone could inhibit sphere formation efficiency, while together with gefitinib treatment, the inhibitory degree seemed to be much higher (Fig. 3E). Therefore, these findings demonstrate that GPX2 deletion suppresses the stem cell-like characteristics of EGFR-TKI resistant cells.

### GPX2 promotes cancer stemness by activating the Sonic Hedgehog signaling pathway

**Fig. 4:**
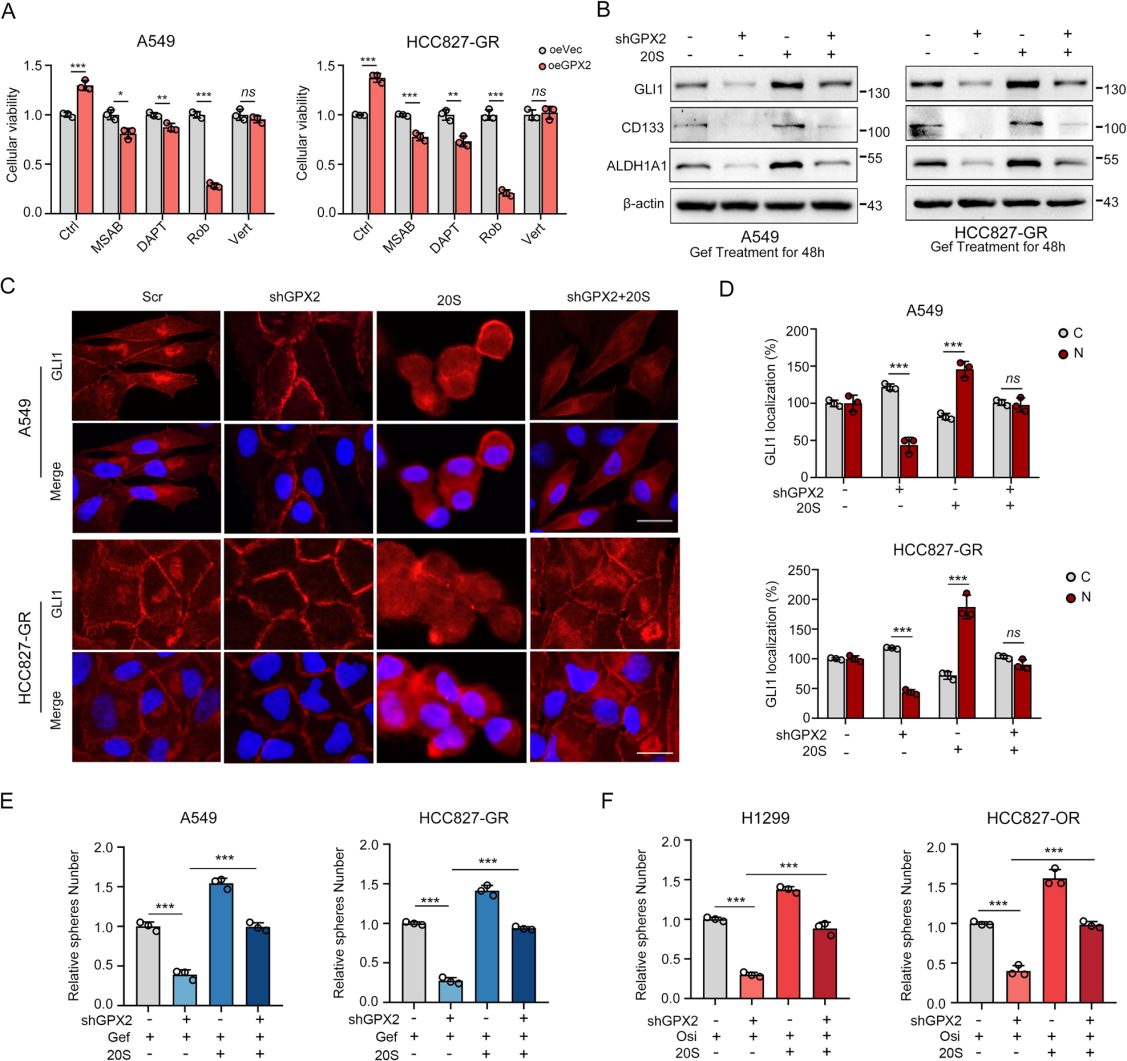
GPX2 promotes cancer stemness by activating the Sonic Hedgehog signaling pathway. **A** The effect of different signaling pathway inhibitors on cellular viability. Rob, Robotnikinin; Vert, Verteporfin. **B** GLI1, CD133 and ALDH1A1 expression after addition of 20(S)-OHC in GPX2 knockdown cells. **C, D** Immunofluorescence staining of GLI1 distribution after replenishing 20(S)-OHC in scramble or GPX2 shRNA transfected A549 and HCC827-GR cells. Scale bar, 20 µm. 20(S)-OHC treatment rescued the decline in the sphere formation efficiency led by the combination of GPX2 shRNA with gefitinib (**E**) or osimertinib (**F**). *P < 0.05, **P < 0.01, ***P < 0.001, ns no significance.

To figure out how GPX2 contributes to characteristics of CSCs and drug tolerance, we treated GPX2-overexpressed cells with different inhibitors of stemness-related signaling pathways, including Wnt pathway (MSAB), Notch pathway (DAPT), Hedgehog pathway (Rob) and Hippo pathway (Verteporfin). The results of the CCK-8 assay revealed that, except for Hippo, these inhibitors could mitigate the growth-promoting effect of GPX2 on tumor cells to varying degrees. Among them, the Hedgehog inhibitor Rob repressed the highest (Fig. 4A). Meanwhile, the Hedgehog signaling pathway activator 20(S)-OHC reversed the downregulation of Hedgehog signaling and cancer stemness markers caused by GPX2 knockdown (Fig. 4B). GLI nuclear transport is critical for Hedgehog signaling. Using immunofluorescence, we found that 20(S)-OHC restored GLI nuclear translocation impaired by GPX2 deficiency. (Fig. 4C–D). Likewise, 20(S)-OHC also rescued the decline in sphere-forming efficiency caused by the combination of GPX2 knockdown and gefitinib or the third generation TKI osimertinib (Fig. 4E–F). These findings demonstrate that GPX2 participates in maintaining the stemness of TKI-resistant cells by activating the Sonic Hedgehog signaling pathway.

### GPX2 augments the Sonic Hedgehog pathway via accelerating ROS scavenging

**Fig. 5:**
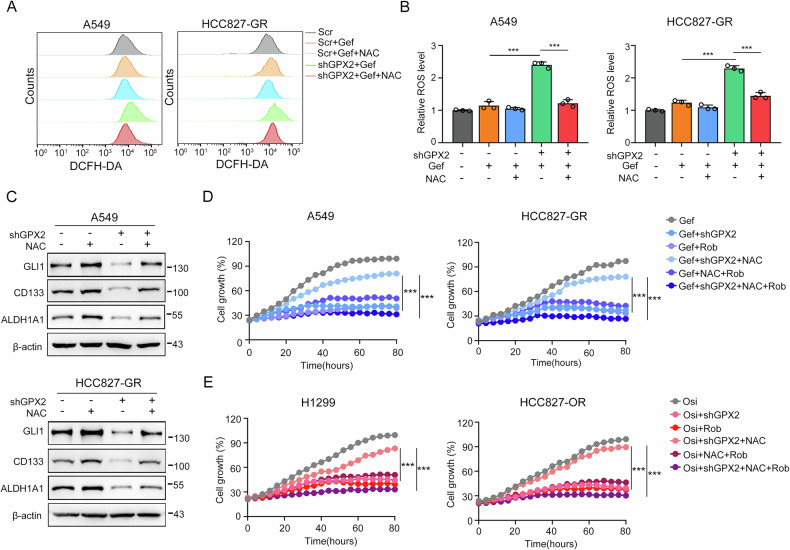
GPX2 augments the Sonic Hedgehog pathway via accelerating ROS scavenging. **A** ROS production in gefitinib-treated A549 and HCC827-GR cells transfected with scramble or GPX2 shRNA in the presence or absence of ROS scavenger (NAC). **B** Quantitative analysis of ROS production. **C** GLI1, CD133 and ALDH1A1 expression in GPX2-knockdown A549 and HCC827GR cell lines treated with NAC. **D** Cell viability was detected for evaluating the effect of supplementation with NAC or Rob on GPX2 silence-induced resensitization to gefitinib in EGFR-TKI resistant cells. **E** The effect of NAC or Rob on GPX2 silence-induced resensitization to osimertinib in EGFR-TKI resistant cells. ***P < 0.001, ns no significance.

ROS have been shown to exert cytotoxic effects but also play a multitude of signaling roles [24]. We hypothesize that GPX2-mediated Hedgehog pathway activation involves ROS scavenging. To test this, cells were treated with the ROS inhibitor N-acetylcysteine (NAC), followed by DCFH-DA staining. The results showed that GPX2 deficiency increased ROS production, while NAC attenuated the ROS accumulation caused by GPX2 knockdown combined with gefitinib (Fig. 5A–B). In consistent with this, protein expressional analysis revealed that NAC reversed the downregulation of Hedgehog signaling and stemness markers under gefitinib treatment in GPX2-knockdown cell lines (Fig. 5C). These data suggest that GPX2 confers EGFR-TKI tolerance by altering ROS levels of cells to modulate Hedgehog signaling transduction. Further real-time cell growth monitoring results also demonstrated that GPX2 inhibition significantly improved the sensitivity to gefitinib of TKI-resistant cell lines, which was eliminated by NAC supplementation (Fig. 5D). In contrast, by using Hedgehog signaling inhibitor Robotnikinin (Rob), we found that it could sensitize resistant cell lines to gefitinib and restore the inhibitory effect of GPX2 intervention on cell growth under NAC treatment (Fig. 5D). The similar results were verified in TKI osimertinib (Fig. 5E). Taken together, GPX2-mediated ROS scavenging is critical for the downstream Hedgehog signaling activation and drug resistance.

### METTL14-mediated m6A modification decreases GPX2 mRNA stability

**Fig. 6:**
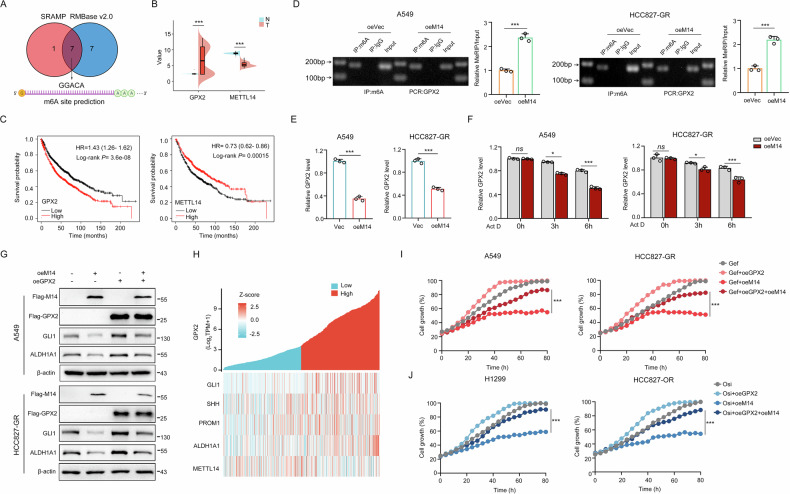
METTL14-mediated m^6^A modification decreases GPX2 mRNA stability in TKI-resistant cells. **A** Predicted m^6^A site in GPX2 mRNA from overlapping results of SRAMP and RMBase v2.0. **B** GPX2 and METTL14 expressional analysis in normal versus tumor lung tissues from TCGA database. **C** Kaplan-Meier analysis of overall survival in lung cancer patients with high or low GPX2/METTL14 expression levels from TCGA database. **D** MeRIP analysis of m^6^A modification on GPX2 in METTL14 overexpressed A549 and HCC827-GR cells. **E** The effect of METTL14 overexpression on GPX2 mRNA level in gefitinib-resistant cell lines. **F** GPX2 expressional analysis in cells overexpressing METTL14 under actinomycin D (5 μg/mL) treatment for 0, 3 and 6 hours. **G** The effect of GPX2 and METTL14 on GLI1 and ALDH1A1 expression in gefitinib-resistant cells. **H** Co-expressional analysis of GPX2 with SHH, GLI1, CD133, ALDH1A1 and METTL14. **I** The effect of GPX2 and METTL14 on gefitinib sensitivity. **J** The effect of GPX2 and METTL14 on osimertinib sensitivity. *P < 0.05, ***P < 0.001, ns no significance.

GPX2 expression is enhanced in gefitinib-resistant cells, but the factor of regulating GPX2 levels remains unclear. Of note, the latest advances in RNA epigenetics underline the role of m^6^A modifications in the resistance to a diversity of chemotherapeutic agents [25]. Here, we used RMBase v2.0 and SRAMP prediction tool and identified seven overlapping m^6^A sites (Fig. 6A). Importantly, Methylated RNA Immunoprecipitation (MeRIP) assay showed higher m^6^A levels of GPX2 mRNA in HCC827 cells compared with A549 cells (Fig. S1A), indicating that m^6^A modification plays an essential role in the expressional control of GPX2.

Gene expression analysis from the TCGA database showed a negative connection between GPX2 and methyltransferases METTL3 and METTL14, while the correlation with METTL14 is more significant (Fig. S1B). Furthermore, GPX2 levels elevated in tumor tissues compared with normal tissues, while METTL14 levels declined (Fig. 6B). Kaplan-Meier survival analysis also showed that patients with higher GPX2 expression or lower METTL14 expression all had a worse overall survival (Fig. 6C). All these results suggest that METTL14 is correlated with GPX2-mediated resistance. By performing MeRIP analysis, we found the cells with METTL14 overexpression displayed higher m^6^A levels of GPX2 mRNA compared to control cells (Fig. 6D). Indeed, overexpressed methyltransferase METTL14 decreased the level of GPX2 (Fig. 6E). In addition, using the mRNA synthesis inhibitor Actinomycin D at different time points, we found that METTL14 elevation reduced GPX2 mRNA levels, indicating that METTL14-mediated m^6^A modification affects GPX2 mRNA stability. (Fig. 6F). GPX2 overexpression reversed the METTL14-induced reduction in Hedgehog pathway activity and stem cell markers. (Fig. 6G). Co-expression analysis in lung cancer tissues also confirmed that GPX2 was positively correlated with Hedgehog signaling and cancer stem cell markers while negatively correlated with METTL14 (Fig. 6H). Real-time cellular analysis showed that the resensitization effect of METTL14 on EGFR-TKI resistance was offset by overexpression of GPX2 (Fig. 6I–J). Overall, these results illustrate that METTL14-mediated m^6^A modification controls GPX2 mRNA expression to affect stemness signaling and drug resistance.

### Targeting GPX2 retards EGFR-TKI resistance in vivo

**Fig. 7:**
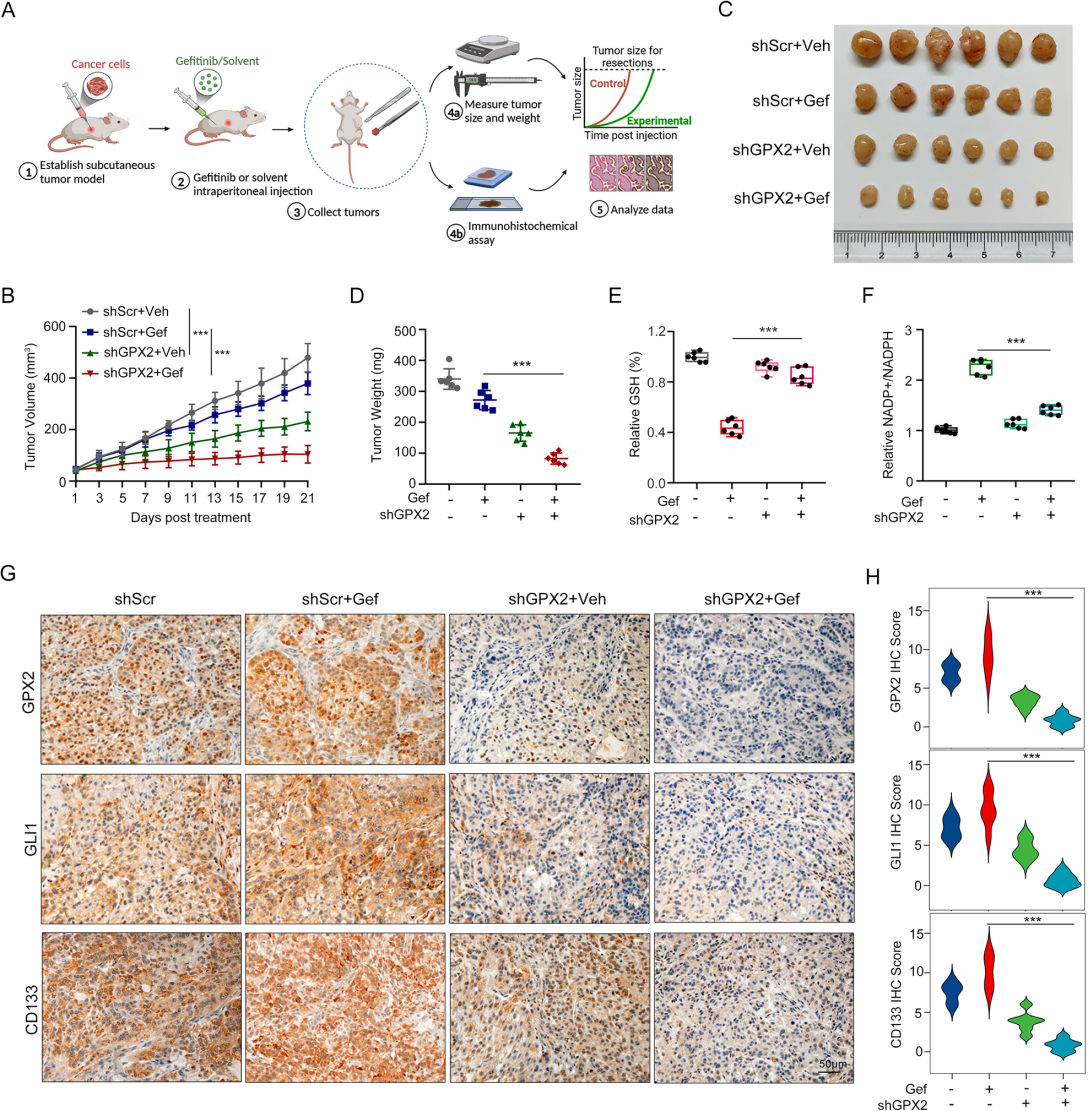
Targeting GPX2 mitigates EGFR-TKI resistance in vivo. **A** Schematic overview illustrating the establishment and treatment of CDX model. **B** Tumor size was measured every 2 days. **C** Representative images of tumors tissues from the CDX model. **D** Tumor weight of each group at the end point. GSH level (**E**) and NADP + /NADPH ratio (**F**) of tumor tissues in different treatment groups were detected. **G** Immunohistochemical staining of GPX2, GLI1 and CD133. Scale bar, 50 µm. **H** Scoring for immunohistochemical staining was calculated. ***P < 0.001.

For evaluating the in vivo role of GPX2 on the efficacy of TKI, we employed CDX models. A549 cells expressing scramble shRNA or GPX2 shRNA were implanted subcutaneously in BALB/c nude mice, which were administered intraperitoneally with a gefitinib dose of 25 mg/kg/day for 21 days (Fig. 7A). Tumor growth measurement demonstrated that GPX2 knockdown shrank the tumor size, while together with gefitinib made the tumor size diminish much more (Fig. 7B). Moreover, combination of GPX2 intervention and gefitinib significantly reduced tumor weight as compared with treatment of gefitinib alone (Fig. 7C–D). To validate these findings, we constructed the HCC827-GR-derived xenograft model. Consistent with the A549-derived results, tumor growth measurement revealed that GPX2 silencing potentiated gefitinib-induced tumor growth inhibition in HCC827-GR-derived nude mice (Fig. S2A), with tumor sizes and endpoint weights demonstrating a synergistic effect of GPX2 knockdown plus gefitinib (Fig. S2B–C). These results demonstrated that loss of GPX2 could restrain tumor growth and alleviate EGFR-TKI resistance in vivo.

To further explore whether GPX2 has effects on glutathione metabolism and cancer stemness in vivo, we performed analysis of reducing equivalents and immunohistochemical staining. In GPX2 deficient group, GSH level and NADP + /NADPH ratio in tumors were not appreciably altered after gefitinib treatment compared to control group (Fig. 7E–F), implying that silencing GPX2 expression impedes cellular utilization of GSH for redox homeostasis. Meanwhile, immunohistochemical and immunoblot analysis showed impaired expression of GLI1 and CD133 in the GPX2 deficient group after gefitinib treatment (Fig. 7G–H, Fig. S2D). Taken together, these findings suggest that targeting GPX2 may be an attractive pharmaceutical intervention for advanced NSCLC and gefitinib-resistant NSCLC patients.

## Discussion

Despite the evolution of EGFR mutation-targeting tyrosine kinase inhibitors (TKIs) through several generations, clinical challenges persist, including primary and acquired resistance, as well as a lack of response to therapy in EGFR wild-type cases. Resistance has developed to both first- and third- generation inhibitors, but the underlying mechanisms remain unclear [25]. Our research has uncovered a pivotal finding that glutathione metabolism within the antioxidant pathway in drug-resistant cell strains is significantly enhanced. Moreover, inhibiting glutathione metabolism or directly targeting key enzyme GPX2 in its metabolic process can effectively improve the therapeutic efficacy of TKIs. This discovery offers new strategies for overcoming resistance in EGFR-mutated tumors and may have a profound impact on future personalized medicine and precision therapeutics.

Cancer cells are typically subjected to higher levels of oxidative stress than normal cells to bolster their rapid development [26, 27]. Their sustained redox adaptation promotes invasive traits and therapy resistance, yet excessive pro-oxidants can trigger cell death [28, 29]. While ROS-based therapies have shown promising results in clinical trials, several barriers persist that limit their therapeutic efficacy. A key challenge is the neutralization of ROS by cellular antioxidant systems, particularly the GSH redox system [27]. The GPX family comprises a huge group of enzymes that prevent the formation of free radicals from hydroperoxides by using GSH as a substrate to reduce them [30]. As an indispensable antioxidant enzyme of GSH metabolism, GPX2 maintains low intracellular ROS level and maintain the clonogenic and metastatic tumor cell population [31]. However, the relation between it and targeted drug resistance in NSCLC and the underlying pharmacodynamic advantages deserve deliberation. Here, we found for the first time that GPX2 was upregulated in EGFR-TKI resistant NSCLC cells and tissues, accelerating the elimination of ROS and conferring drug resistance in NSCLC.

Increasing evidence suggests that acquired resistance to EGFR-TKIs is associated with an enhanced cancer stem cell (CSC) phenotype [32]. Key signaling pathways governing cancer stemness, including the Wnt [33], Notch [34], and Hedgehog [35], are known to regulate resistance to EGFR-TKIs. In our study, we observed that the knockdown of GPX2 led to the downregulation of GLI1, a key target gene of the Hedgehog signaling pathway, and reduced the sphere-forming efficiency in TKI-resistant cells, indicating that GPX2 plays a crucial role in maintaining the stemness and resistance phenotype. Given that CSCs exhibit lower ROS compared to their non-tumorigenic counterparts [36–38], we treated cells with ROS scavenger NAC and found that it could counteract the Hedgehog signaling alterations induced by GPX2 inhibition. This suggests that GPX2 upregulation in TKI-resistant cells may enhance the antioxidant response, reduce ROS accumulation, and promote Hedgehog pathway activation, along with associated stemness and resistance phenotypes. However, the mechanism by which GPX2-mediated ROS reduction activates the Hedgehog pathway remains to be elucidated.

N6-methyladenosine is the predominant post-transcriptional modification in eukaryotic mRNA and regulates translation, metabolism, and stability [39]. The dysregulation of m^6^A modification has been implicated in modulating malignant behaviors such as tumor stemness and drug resistance [40, 41]. For instance, reduced m^6^A levels enhances the therapeutic efficacy of crizotinib, an ALK/ROS1/c-MET kinase inhibitor, in the treatment of non-small cell lung cancer (NSCLC) with high c-MET expression [42]. While the overexpression of METTL3, a key m^6^A methyltransferase, boosts sorafenib sensitivity in hepatocellular carcinoma by stabilizing FOXO3 mRNA via m^6^A methylation [43]. These observations indicate that the impact of m^6^A modification on drug resistance varies by cancer-type and context-specific. Here, we demonstrated a negative correlation between METTL14 and GPX2. METTL14-mediated m^6^A modification reduces the stability of GPX2 mRNA, which in turn inhibits stemness signaling and confers drug resistance. This discovery provides a deeper understanding of the intricate role of m^6^A modification in the development of drug resistance and suggests potential therapeutic targets for overcoming resistance in cancer treatment.

Here, we demonstrate that glutathione peroxidase GPX2 confers resistance to EGFR-targeted therapies in both EGFR wild-type and mutant NSCLC cell lines by enhancing ROS-scavenging. The resulting ROS reduction activates the Hedgehog pathway, thereby promoting cancer stemness. Notably, increased m^6^A methylation on GPX2 mRNA correlates with reduced mRNA stability and lower GPX2 expression. Using CDX models, we confirmed that suppressing GPX2 expression significantly enhances in vivo therapeutic efficacy of gefitinib. These findings highlight GPX2 as a promising target to improve gefitinib response in NSCLC treatment.

## Materials and methods

### Cell culture

The human NSCLC cell lines A549 and HCC827 were purchased from Cell Bank of Chinese Academy of Sciences (Shanghai, China) and incubated at 37 °C with 5% CO_2_. The cells were grown in RPMI-1640 medium (Sigma-Aldrich, R6504) with 10% FBS (Gibco, 16140071) and 1% penicillin-streptomycin (Gibco, 15140-122). To establish the acquired EGFR-TKI resistant cell line, HCC827 cells were exposed to increasing concentrations (from 1 μM to 5 μM) of gefitinib (Selleck Chemicals, S1025) for 6 months. A final concentration of 1 µM gefitinib was used to maintain HCC827-GR cells. The absence of Mycoplasma was confirmed on all cell lines. Cell viability was examined by CCK-8 each time to confirm the acquired resistance of cells.

### GSH measurement

The intracellular GSH level was measured by using the GSH and GSSG assay kit (Beyotime, S0053) according to the manufacturer’s instructions. The samples were resuspended in Protein Removal Reagent M and underwent freeze-thaw cycles before centrifugation. For GSSG, samples were treated with GSH Scavenger. The absorbance was detected at OD412 nm via BioTek Gen5 system (BioTeck, VT, USA), and GSH level was calculated as follows: GSH = Total Glutathione - GSSG × 2.

### NADP + /NADPH quantitation

The intracellular NADP + /NADPH ratio was measured using NADP + /NADPH Assay Kit with WST-8 (Beyotime, S0179). All experimental steps were well according the manufacturer’s instruction. The NADPH signal intensity was detected at OD450 nm via BioTek Gen5 system (BioTeck, VT, USA). The NADP + /NADPH ratio was calculated as: [NADP + ]/[NADPH] = ([NADP_total_] - [NADPH])/[NADPH]

### ROS detection

Intracellular ROS levels were quantified by using the fluorescent oxidation indicator DCFH-DA (Beyotime, S0035S) according to the manufacturer’s instructions. Briefly, 1 × 10^6^ cells were inoculated in 6-well plates, incubated with 10 μM DCFH-DA at 37 °C for 20 minutes in an incubator away from light, and washed gently with PBS. BD FACSCelesta Flow Cytometer (BD Biosciences, NJ, US) was used to measure fluorescence intensity.

### Human specimens

12 TKI-responsive and 8 TKI non-responsive tumor tissues were obtained by lung biopsy from patients undergoing neoadjuvant chemotherapy in the Third Hospital of the Naval Medical University, Shanghai, China. The diagnosis of lung cancer was confirmed by pathologic examination of histopathological slides. The study was approved by the Committee on Ethics of Medicine of Naval Medical university. All subjects who donated samples signed an informed consent.

### Immunohistochemical (IHC) analysis

The lung cancer tissue slides were constructed from formalin-fixed, paraffin-embedded specimens and IHC staining was performed according to UltraVision Quanto Detection protocols (Thermo Scientific, 34065). The photographs were obtained by Nikon eclipse Ti-s microscope (Nikon, Tokyo, Japan). Briefly, the score of the extent of the IHC staining area of the sections was set from 0 to 4. The intensity of IHC was scored from 0 to 3. The extent score and intensity score were multiplied to reach the final score that was used for the analysis.

### Electric Cell-Substrate Impedance Sensing (ECIS)

Real-time analysis of cell proliferation was performed using the ECIS model 9600 (Applied Biophysics, NY, USA). In brief, the procedure began by functionalizing 96W20idf ECIS electrode array with 10 mM cysteine at 37 °C for 2 hours. Cells were seeded into chambers at a density of 4 × 10^4^ cells per well and allowed to settle for 20 minutes to form a monolayer. Subsequently, cells were subjected to various treatments according to experimental protocols, and real-time changes in cell impedance were recorded. Data analysis was performed using the CP96 Analyze software.

### Sphere-formation assay

Cells were inoculated at a density of 100 cells/well in 96-well ultra-low adsorption microtiter plates (Corning, 3474). Cells were cultured in serum-free RPMI-1640 medium supplemented with B27 (ThermoFisher Scientific, 17504044), 20 ng/ml EGF (MedChemExpress, P7109), 4 μg/mL heparin (Stem Cell Technologies, 07980), and 20 ng/mL basic fibroblast growth factor (MedChemExpress, P7004) for 14 days. The spheres were photographed and numbered using Nikon eclipse Ti-s microscope (Tokyo, Japan).

### Methylated RNA Immunoprecipitation (MeRIP)

Total RNA was extracted using TRIzol (Thermo Fisher Scientific, 15596026CN). RNA was randomly fragmented by using NEBNext® Magnesium RNA Fragmentation Modul (NEB, E6150S). Anti-IgG and anti-m^6^A (Cell Signaling Technology, 56593) antibodies were conjugated to Protein G-Agarose beads (Roche, 11243233001) in the reaction buffer for 4 hours at 4 °C. Fragmented mRNA was then added to the mixture and incubated for 16 hours at 4 °C with slow tumbling. The RNA was washed three times with IPP buffer (10 mM Tris-HCl, pH 7.4, 150 mM NaCl, 0.1% NP-40), digested with proteinase K, and then isolated using TRIzol. Changes in m^6^A methylation of target genes were measured by real-time qPCR following m^6^A-IP (Table S2).

### RNA stability assays

Actinomycin D (Sigma, SBR00013) was administered to NSCLC cells for 0, 3 and 6 hours. GPX2 mRNA levels were quantified using real-time qPCR. The mRNA expression of each group at different time points was calculated and normalized by β-Actin. The rate of GPX2 mRNA degradation (K_decay_) is formulated as follows:

Ln (C/C0)=-K_decay_t

In the formula, t represents the transcriptional inhibition time, and C represents the mRNA level at time t. C0 is the mRNA level at 0 hour. Hence, the mRNA half-time (t_1/2_) is calculated as follows:

Ln (1/2)=-K_decay_t_1/2_

### In vivo cell-derived xenograft assays

5-week-old male BALB/c nude mice from SLAC (Shanghai, China) were divided into four groups at random. A549 or HCC827-GR cells stably transfected with Scramble shRNA and GPX2 shRNA were resuspended in phosphate-buffered saline, then 5 × 10^6^ A549 or 3 × 10^6^ HCC827-GR cells were injected subcutaneously into each nude mouse. Seven days after inoculation, gefitinib was administered intraperitoneally daily at a dose of 25 mg/kg, with 1% Tween 80 (Sigma, P4780) in physiological saline water serving as the control. The growth of the tumor was monitored every two days using electronic equipment. The tumor volume (mm^3^) was calculated as (length × width^2^) × π/6. The animals were sacrificed after 28 days for removing the tumors. 4% paraformaldehyde was used to fix tumor tissues for immunohistochemistry.

### Statistical analysis

Each experiment was conducted independently three times and the results were presented as the mean ± SD. SPSS V.22.0 and GraphPad Prism software were used to analyze all of the data. Survival probability was determined using the Kaplan-Meier method, and differences were assessed using the log-rank test. The analysis of variance (ANOVA) followed by the Tukey multiple comparisons test or two-tailed Student’s *t* test was used for the statistical analysis.

## Supplementary information

Supplemental information

WB source data

## Data Availability

All data generated or analyzed during this study are included either in this article or in the supplementary information files.
