# Supplementary material for: N6-methyladenosine-modified GPX2 impacts cancer cell stemness and TKI resistance through regulating of redox metabolism
Source: Cell Death Dis. 2025 Jun 18;16(1):458. doi: 10.1038/s41419-025-07764-0 (PMC12177039; doi:10.1038/s41419-025-07764-0)
Supplement: Supplementary file 1 — Supplemental information [file 41419_2025_7764_MOESM1_ESM.docx]

**N6-methyladenosine-modified GPX2 impacts cancer cell stemness and TKI resistance through regulating of redox metabolism**

Appendix Methods

Table S1 Primer sets used in RT-qPCR experiments.

Table S2 Primer sets used in PCR experiments.

Table S3 List of target sequences against shRNAs.

Figure S1. The relationship between m6A modification and GPX2.

Figure S2 Targeting GPX2 mitigates EGFR-TKI resistance in the HCC827-GR-derived xenograft model.

**Appendix Methods**

**Immunofluorescence**

Cultured cells on glass slides were fixed with 4% paraformaldehyde for 30 minutes at room temperature, permeabilized with 0.1% Triton X-100 for 5 minutes, and then blocked with 3% BSA for 1 hour. After overnight incubation at 4°C with anti-ALDH1A1 primary antibody (Proteintech, 15910-1-AP), the cells were stained with anti-rabbit Alexa Fluor 488 secondary antibody (Abcam, ab150077) for 1 hour. DAPI was applied for nuclear staining for 15 minutes. Coverslips were mounted on the slides using an anti-fluorescence quenching sealer. Laser confocal microscopy (Leica SP8, Wetzlar, Germany) was used for the acquisition and analysis of images.

**Immunoblot**

Equal amounts of protein lysate were separated on polyacrylamide gels and transferred onto polyvinylidene difluoride membranes (Millipore, Billerica, MA, USA). After blocking with 5% skim milk powder in TBST for 2 h, the membranes were incubated with specific antibodies against GPX2 (Signalway Antibody, 29533), ALDH1A1 (Proteintech, 15910-1-AP), CD133 (Proteintech, 18470-1-AP), GLI1 (Cell Signaling Technology, 3538) and FLAG (Cell Signaling Technology, 14793) at appropriate dilutions. The images were analyzed using the Tanon 5200 image analyzer (Tannon, Shanghai, CN) after secondary antibodies.

**CCK-8 assay**

NSCLC cells viability was assessed by Cell Counting Kit 8 (Beyotime, C0037). In short, NSCLC cells were seeded in 96-well plates at a density of 4000-5000 cells per well. After different treatments, cells were incubated for 1h with CCK-8 reagent and the absorbance was measured at OD450 nm using a BioTek Gen5 system (BioTeck, VT, USA). All experiments were performed three times independently.

**RNA extraction, reverse transcription and Real-time quantitative PCR (RT-qPCR) analysis**

Total RNA was extracted using TRIzol (Thermo Fisher Scientific, 15596026CN). RNA was then reverse-transcribed into cDNA using the PrimeScript RT reagent Kit (Takara, RR037Q) following the manufacturer’s instructions. The expression levels of the target RNAs were quantified by real-time PCR using SYBR Premix Ex Taq (TaKaRa, RR390Q) and normalized to β-Actin. The primer sequences for this study are listed in Table S1. The relative expression of mRNA was analyzed based on the ΔΔCT method.

**Plasmids construction, lentiviral shRNAs and transfections**

GPX2 and METTL14 were amplified by PCR and constructed into the pcDNA3.1- 3xFlag vector (Invitrogen, CA, USA). The lentiviral shRNAs specifically targeting GPX2 were synthesized by Genomeditech (Shanghai, China). Targeting sequences are listed in supplementary Table S3. Plasmids were transfected into cells using Lipofectamine 3000 (Thermo Fisher Scientific, L3000001) following the manufacturer’s instructions.

**Table S1-S3**

**Table S1 Primer sets used in RT-qPCR experiments.**

| **Gene** | **Accession number** | **Sequences (5' to 3')** |
| --- | --- | --- |
| Human *GPX2* | NM_002083.4 | Forward: AGAATGTGGCTTCGCTCTGA  Reverse: CCTCATTCTGACAGTTCTCCTGA |
| Human *GPX8* | NM_001008397.4 | Forward: CTAGGATCTGAAGGAGAACCTGC  Reverse: GCCTGATGACTTCAATGGGCTC |
| Human *GSTK1* | NM_001143679.2 | Forward: AAGCTCCTGAGACACCATCTCC  Reverse: CTCTGGATGCTCCAAGTTCACG |
| Human *GSTM2* | NM_001142368.2 | Forward: AGATCACCCAGAGCAACGCCAT  Reverse: GGCTGTCCATAAACTGGTTCTCC |
| Human *GSTM4* | NM_000850.5 | Forward: TGGAGAACCAGGCTATGGACGT  Reverse: CCAGGAACTGTGAGAAGTGCTG |
| Human *IDH1* | NM_005896.4 | Forward: CTATGATGGTGACGTGCAGTCG  Reverse: CCTCTGCTTCTACTGTCTTGCC |
| Human *METTL14* | NM_020961.4 | Forward: CTGAAAGTGCCGACAGCATTGG  Reverse: CTCTCCTTCATCCAGATACTTACG |
| Human *ACTB* | NM_001101.5 | Forward: CACCATTGGCAATGAGCGGTTC  Reverse: AGGTCTTTGCGGATGTCCACGT |

**Table S2 Primer sets used in PCR experiments.**

| **Gene** | **Accession number** | **Sequences (5' to 3')** |
| --- | --- | --- |
| Human *GPX2* | NM_002083.4 | Forward: GGTAGATTTCAATACGTTCCGGG  Reverse: TGACAGTTCTCCTGATGTCCAAA |

**Table S3 List of target sequences against shRNAs.**

| **Gene** | **Sequences (5' to 3')** |
| --- | --- |
| Human *Scramble* | TTCTCCGAACGTGTCACGT |
| Human *GPX2*#1 | CCGATCCCAAGCTCATCATTT |
| Human *GPX2*#2 | GGCTTCCCTTGCAACCAATTT |
| Human *GPX2*#3 | GCACCTTCCCAACCATCAACA |


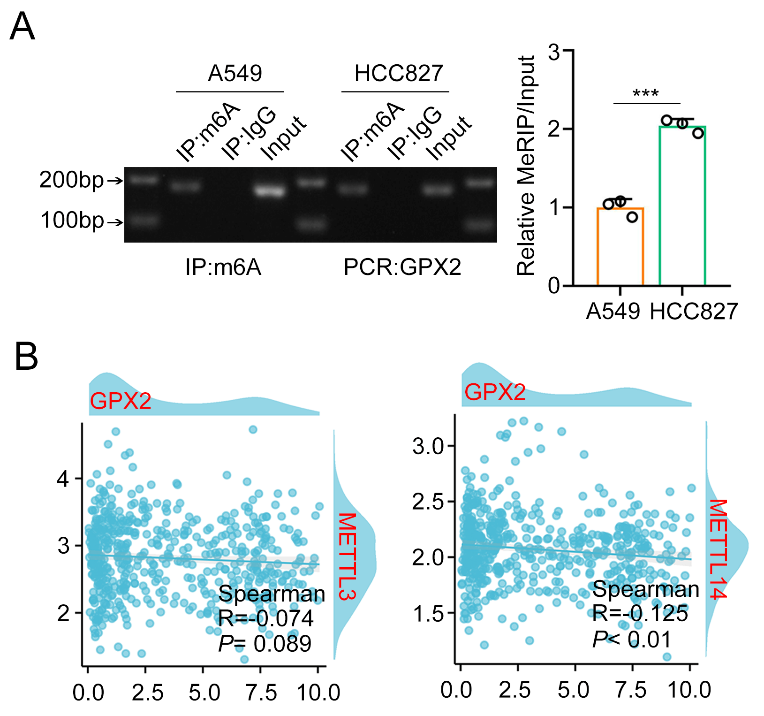


**Figure S1.** **The relationship between m^6^A modification and GPX2.** (**A**) MeRIP assays for m^6^A-modified GPX2 mRNA in A549 and HCC827 cells. (**B**) Scatter plot showing the correlation analysis between GPX2 and METTL3 or METTL14 in the TCGA dataset.

**
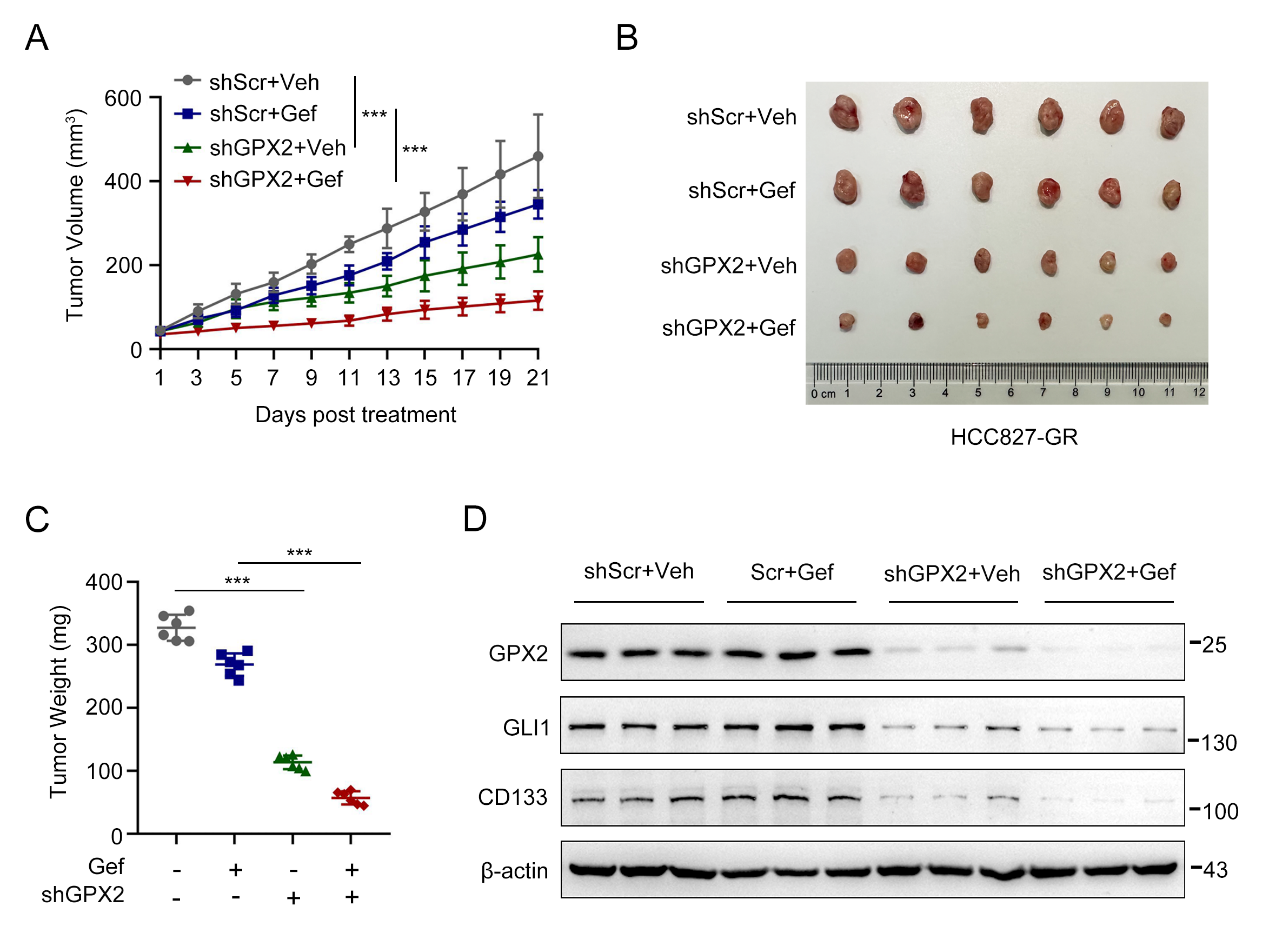
**

**Figure S2.** **Targeting GPX2 mitigates EGFR-TKI resistance in the HCC827-GR-derived xenograft model.** (**A**) Tumor size was measured every 2 days. (**B**) Representative images of tumors tissues from the CDX model. (**C**) Tumor weight of each group at the end point. (**D**) The protein expression of GPX2, GLI1 and CD133.
