# Supplementary figures and images for: N6-methyladenosine-modified GPX2 impacts cancer cell stemness and TKI resistance through regulating of redox metabolism
Source: Cell Death Dis. 2025 Jun 18;16(1):458. doi: 10.1038/s41419-025-07764-0 (PMC12177039; doi:10.1038/s41419-025-07764-0)

**Fig2:**

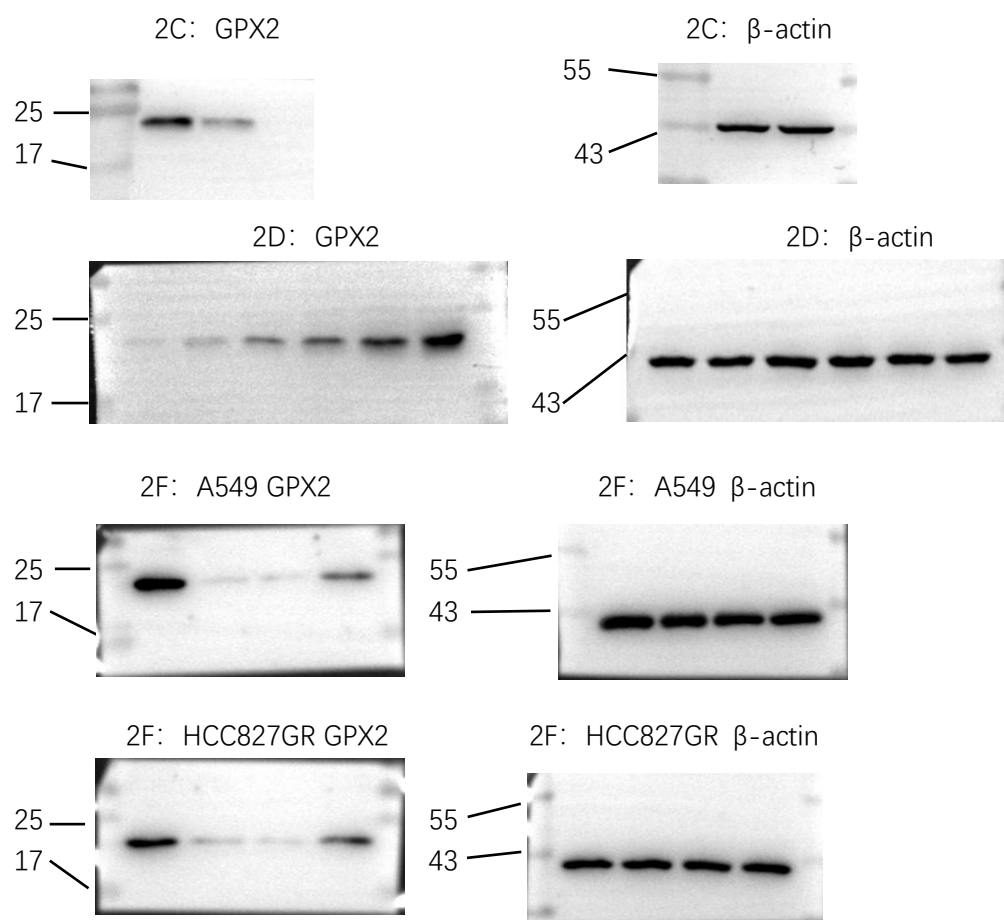

**Fig3:**

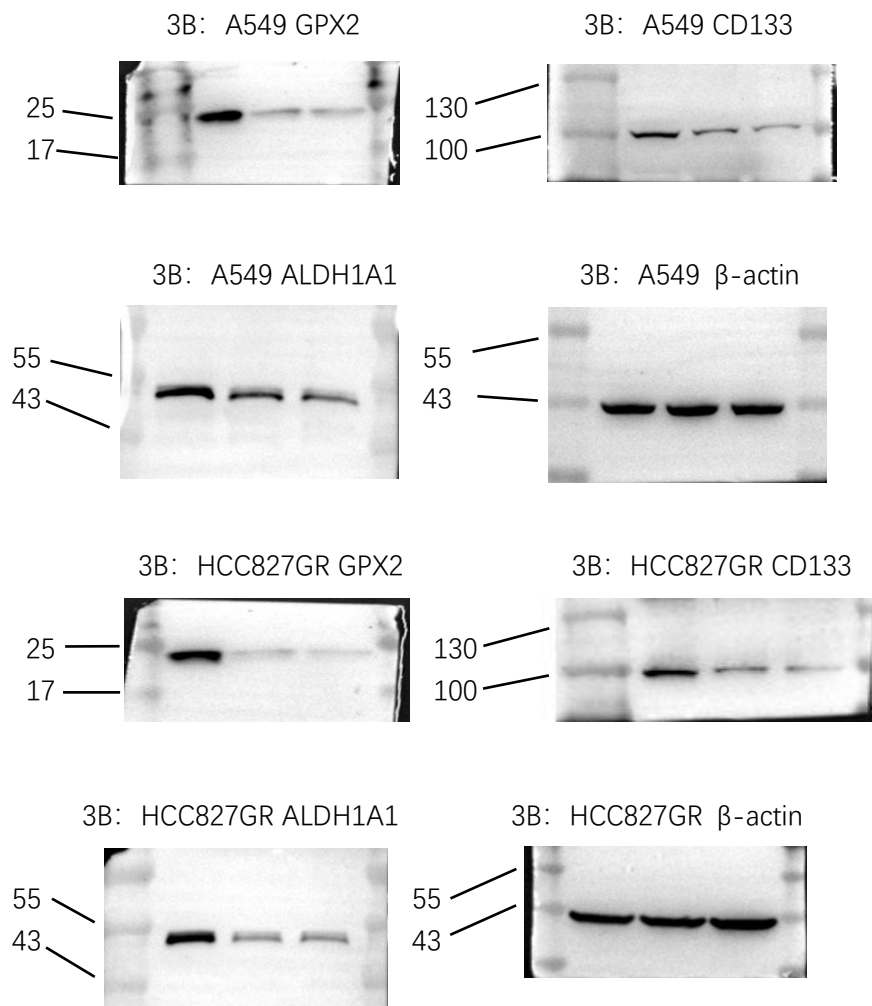

**Fig4:**

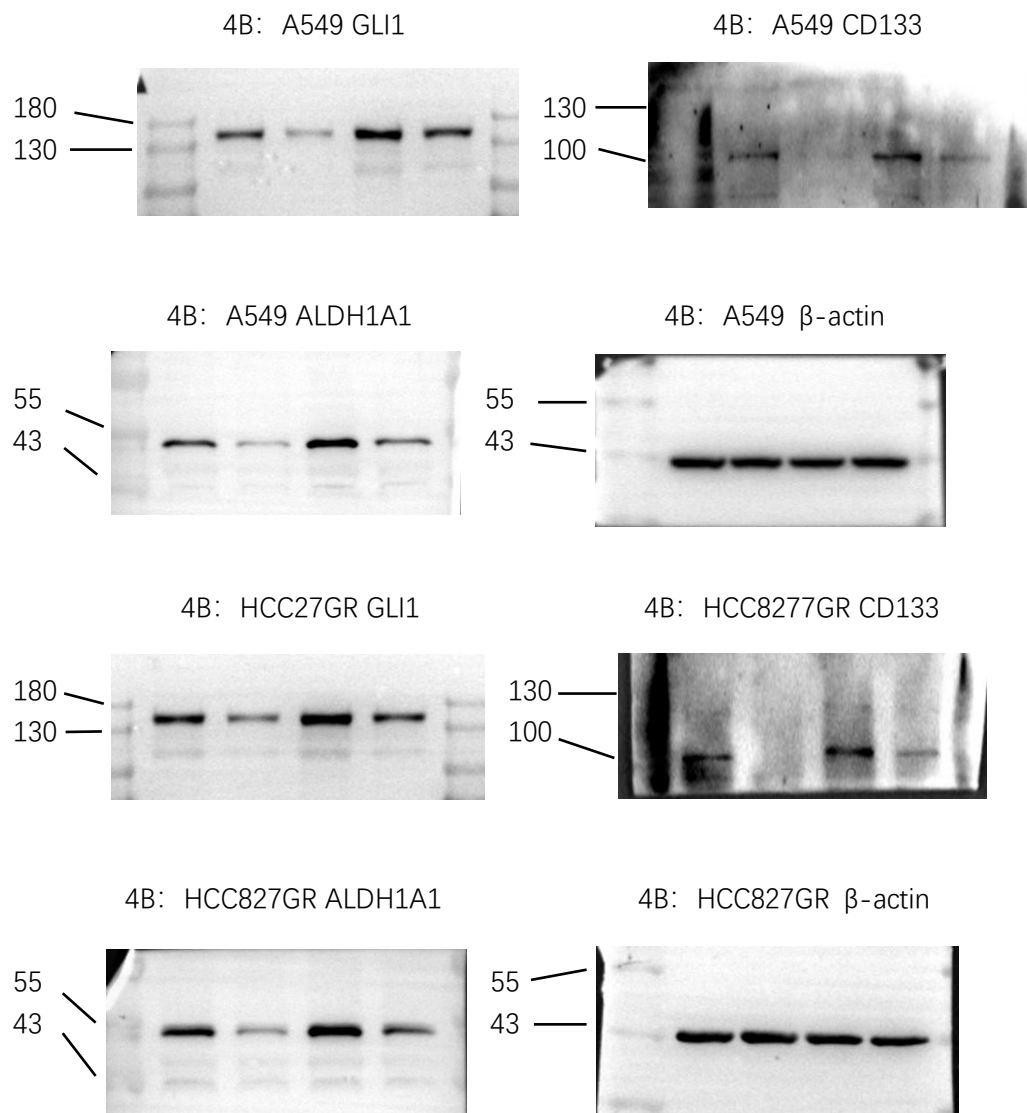

**Fig5:**

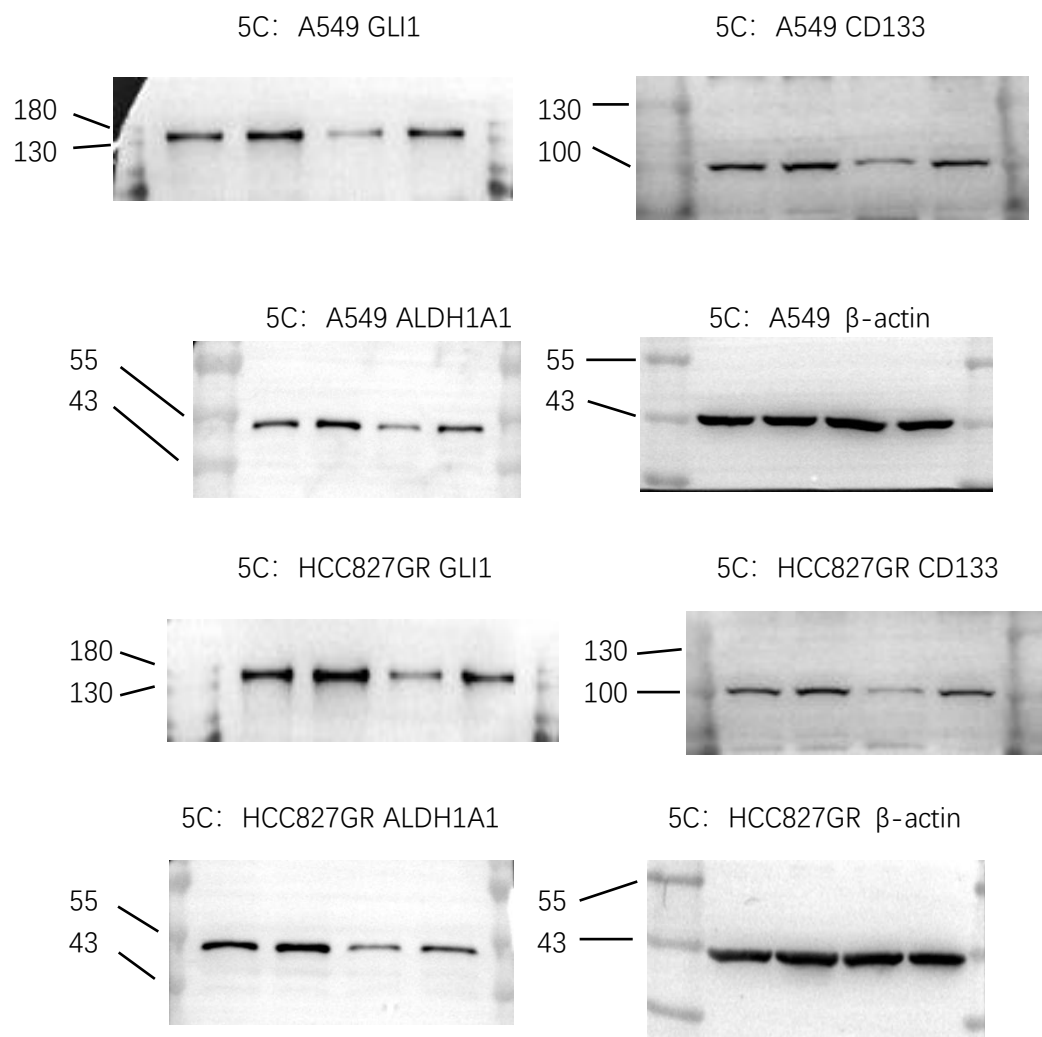

**Fig6:**

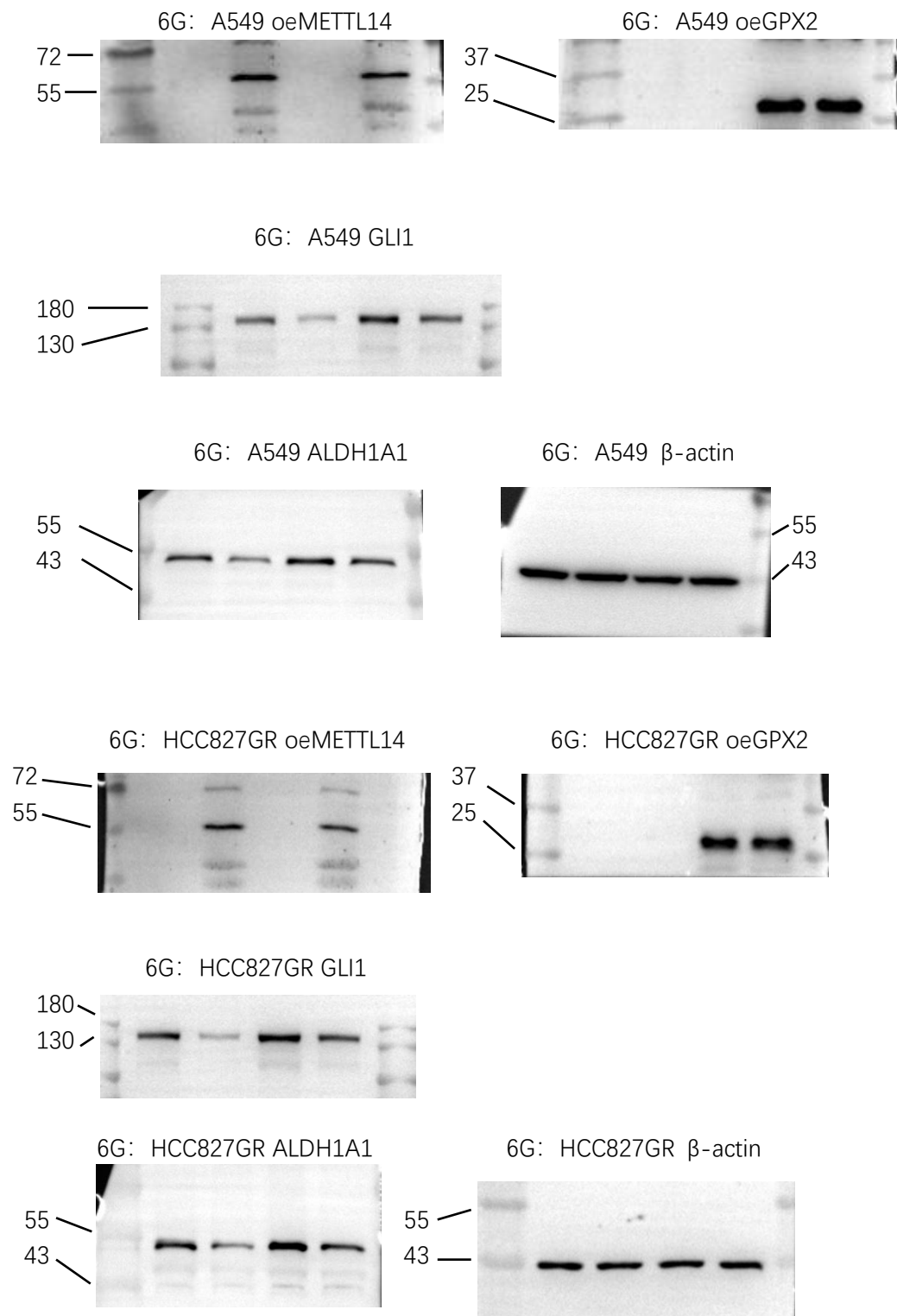

**FigS2:**

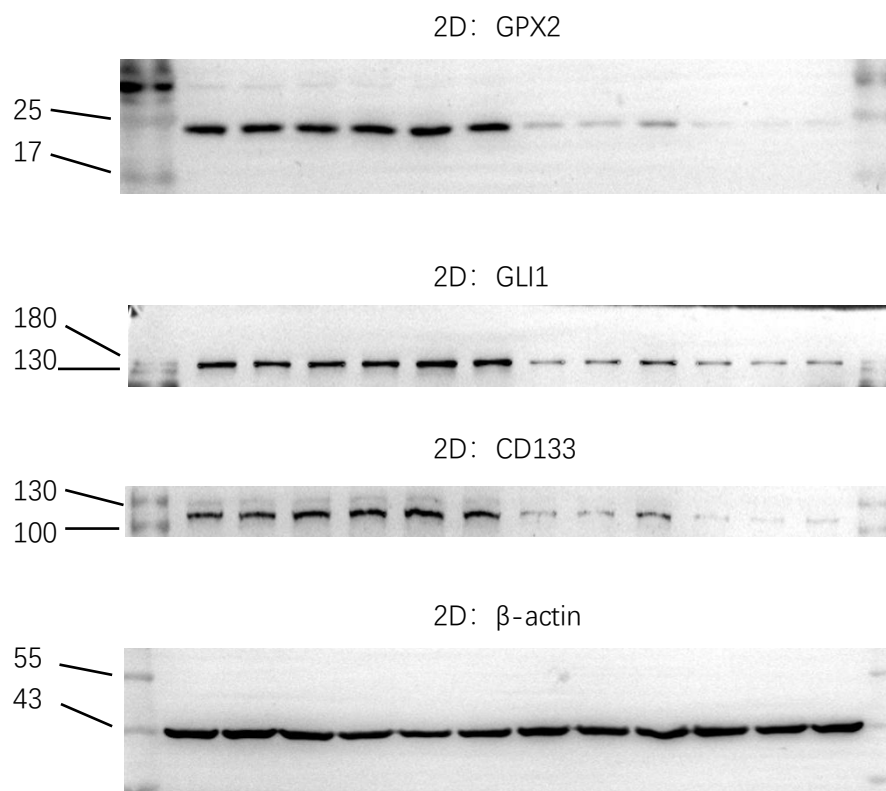

Supplement: Supplementary file 2 — WB source data [file 41419_2025_7764_MOESM2_ESM.pdf]
